# What cancer is teaching us about cellular metabolism

**DOI:** 10.1186/2049-3002-2-S1-O32

**Published:** 2014-05-28

**Authors:** Craig B Thompson

**Affiliations:** 1 Memorial Sloan-Kettering Cancer Center, New York, NY, USA

## 

Past metabolic studies have focused on terminally differentiated cells. These studies suggested that cells maintain bioenergetics through cell-autonomous uptake of available extracellular nutrients, including glucose, lipids, and amino acids, and feeding the catabolites of these nutrients into the TCA cycle. However, recent studies using different mammalian cell types have led to the discovery that mammalian cells lack the cell autonomous ability to take up nutrients. When cultured in the absence of receptor ligands, mammalian cells lack the ability to take up sufficient nutrients to sustain ATP synthesis. In mammalian cells the uptake of extracellular nutrients is directed by growth factor signaling.

Cellular dependence on ligand-directed nutrient uptake establishes a mechanism by which all cells in multicellular organisms might be rendered dependent on receptor dependent signals for survival. In addition, the receptors that direct nutrient uptake, when maximally stimulated, can direct a cell to take up glucose in excess of its needs. The major oncogenic determinants of glucose uptake are receptor tyrosine kinases, PI3K, Akt, and TOR. Their activation results in the conversion of intermediate metabolism to aerobic glycolysis (the Warburg Effect). The net effect of this metabolism is a diversion of available amino acids into protein synthesis and the establishment of an alternative mitochondrial/ cytoplasmic citric acid cycle that converts glucose into lipids.

Although cell growth can be supported effectively by aerobic glycolysis, the conversion from growth to proliferation relies on a cell’s ability to switch from glucose to glutamine for the support of bioenergetics. The oncogene myc has recently been implicated in this conversion. This conversion allows glucose metabolites to be redirected into the pentose phosphate shunt and into serine biosynthesis to support nucleotide production and the maintenance of cellular redox.

A role for intracellular metabolites in oncogenic signaling and cellular differentiation has also been recently suggested through studies of succinate dehydrogenase and fumarase mutations in cancer. The discovery of cancer-associated mutations in isocitrate dehydrogenase 1 and 2 that produce a novel metabolite, 2-hydroxyglutarate, provides the most compelling example of this principle to date. The implications of these recent findings to carcinogenesis and to the development of novel therapeutics for cancer treatment will be discussed.

